# Sequential Exposure to Antenatal Microbial Triggers Attenuates Alveolar Growth and Pulmonary Vascular Development and Impacts Pulmonary Epithelial Stem/Progenitor Cells

**DOI:** 10.3389/fmed.2021.614239

**Published:** 2021-02-22

**Authors:** Helene Widowski, Niki L. Reynaert, Daan R. M. G. Ophelders, Matthias C. Hütten, Peter G. J. Nikkels, Carmen A. H. Severens-Rijvers, Jack P. M. Cleutjens, Matthew W. Kemp, John P. Newnham, Masatoshi Saito, Haruo Usuda, Matthew S. Payne, Alan H. Jobe, Boris W. Kramer, Tammo Delhaas, Tim G. A. M. Wolfs

**Affiliations:** ^1^Department of Pediatrics, Maastricht University Medical Center, Maastricht, Netherlands; ^2^Department of BioMedical Engineering, Maastricht University Medical Center, Maastricht, Netherlands; ^3^GROW School for Oncology and Developmental Biology, Maastricht University Medical Center, Maastricht, Netherlands; ^4^Department of Respiratory Medicine, Maastricht University, Maastricht, Netherlands; ^5^NUTRIM School of Nutrition and Translational Research in Metabolism, Maastricht University Medical Center, Maastricht, Netherlands; ^6^Neonatology, Pediatrics Department, Faculty of Health, Medicine and Life Sciences, Maastricht University Medical Center, Maastricht, Netherlands; ^7^University Children's Hospital Würzburg, University of Würzburg, Würzburg, Germany; ^8^Department of Pathology, University Medical Center Utrecht, Utrecht, Netherlands; ^9^Department of Pathology, Maastricht University Medical Center, Maastricht, Netherlands; ^10^CARIM School for Cardiovascular Diseases, Maastricht University Medical Center, Maastricht, Netherlands; ^11^Division of Obstetrics and Gynecology, The University of Western Australia, Crawley, WA, Australia; ^12^Tohoku University Centre for Perinatal and Neonatal Medicine, Tohoku University Hospital, Sendai, Japan; ^13^Perinatal Institute Cincinnati Children's Hospital Medical Center, Cincinnati, OH, United States; ^14^School for Mental Health and Neuroscience, Maastricht University, Maastricht, Netherlands

**Keywords:** polymicrobial infection, vascular disturbances, adverse pulmonary outcomes, endogenous pulmonary stem cells, bronchopulmonary dysplasia, preterm birth, antenatal inflammation

## Abstract

Perinatal inflammatory stress is strongly associated with adverse pulmonary outcomes after preterm birth. Antenatal infections are an essential perinatal stress factor and contribute to preterm delivery, induction of lung inflammation and injury, pre-disposing preterm infants to bronchopulmonary dysplasia. Considering the polymicrobial nature of antenatal infection, which was reported to result in diverse effects and outcomes in preterm lungs, the aim was to examine the consequences of sequential inflammatory stimuli on endogenous epithelial stem/progenitor cells and vascular maturation, which are crucial drivers of lung development. Therefore, a translational ovine model of antenatal infection/inflammation with consecutive exposures to chronic and acute stimuli was used. Ovine fetuses were exposed intra-amniotically to *Ureaplasma parvum* 42 days (chronic stimulus) and/or to lipopolysaccharide 2 or 7 days (acute stimulus) prior to preterm delivery at 125 days of gestation. Pulmonary inflammation, endogenous epithelial stem cell populations, vascular modulators and morphology were investigated in preterm lungs. Pre-exposure to UP attenuated neutrophil infiltration in 7d LPS-exposed lungs and prevented reduction of SOX-9 expression and increased SP-B expression, which could indicate protective responses induced by re-exposure. Sequential exposures did not markedly impact stem/progenitors of the proximal airways (P63+ basal cells) compared to single exposure to LPS. In contrast, the alveolar size was increased solely in the UP+7d LPS group. In line, the most pronounced reduction of AEC2 and proliferating cells (Ki67+) was detected in these sequentially UP + 7d LPS-exposed lambs. A similar sensitization effect of UP pre-exposure was reflected by the vessel density and expression of vascular markers VEGFR-2 and Ang-1 that were significantly reduced after UP exposure prior to 2d LPS, when compared to UP and LPS exposure alone. Strikingly, while morphological changes of alveoli and vessels were seen after sequential microbial exposure, improved lung function was observed in UP, 7d LPS, and UP+7d LPS-exposed lambs. In conclusion, although sequential exposures did not markedly further impact epithelial stem/progenitor cell populations, re-exposure to an inflammatory stimulus resulted in disturbed alveolarization and abnormal pulmonary vascular development. Whether these negative effects on lung development can be rescued by the potentially protective responses observed, should be examined at later time points.

## Introduction

Perinatal inflammatory stress, including sepsis and mechanical ventilation are strongly associated with adverse pulmonary outcomes after preterm birth (1, 2). One of the most frequently occurring complications after perinatal insults and preterm birth is bronchopulmonary dysplasia (BPD), a chronic respiratory disorder of the premature infant. BPD results from a demand of respiratory support and supplemental oxygen after preterm birth and histologically manifests as a delay in alveolar growth and an impairment in vascular maturation (3, 4). Antenatal infections are an essential factor of perinatal stress and associated with preterm delivery and induction of lung inflammation and injury, thereby pre-disposing to BPD (5, 6).

Recently, we showed that timing of antenatal infection/inflammation and its duration of determine the extent and location of adverse effects in the preterm lungs (7). More precisely, we reported attenuated levels of endogenous stem/progenitor populations and their potential consequences, including altered surfactant protein expression and reduced alveolar differentiation in the course of antenatal inflammation (7).

Antenatal infection is often of polymicrobial nature, with *Ureaplasma* (UP) species being the most frequently cultivated bacteria in human amniotic fluid samples (8). Conceivably, potential interactions between various consecutive inflammatory stimuli might modulate the inflammatory response and either lead to a milder outcome, including a treatable surfactant deficiency, or more severe adverse pulmonary outcome (BPD) in the preterm infant. This concept is supported by earlier findings in different preterm organ systems, showing that sequentially occurring antenatal inflammatory insults of varying exposure time points and durations, cause either preconditioning or sensitization to a consecutive inflammatory hit (9–11). With respect to the lungs, *in utero* sequential exposure to antenatal bacteria and bacteria-derived endotoxins resulted in increased inflammation, along with exacerbation of vascular disturbances in very preterm ovine lungs (12). Conversely, in fetuses of higher gestational age (GA), Kallapur et al. showed that chronic UP exposure pre-conditioned the immature lungs and thereby led to a decreased pro-inflammatory response to a subsequent endotoxin hit (13). The GA of the fetus and the duration of each antenatal insult have been shown to modulate the responsiveness of the lung tissue to inflammation and determine the extent of developmental changes.

Considering the increasing importance of aberrant vascular development in neonatal lung diseases (14), and recent findings of endogenous epithelial stem/progenitor cells playing a key role in the adverse pulmonary development (7, 15), our aim was to examine the consequences of sequential inflammatory stimuli on inflammatory read outs and on these crucial developmental aspects. For this purpose, we used a translational ovine model of antenatal infection/inflammation with consecutive exposures to a chronic and an acute stimulus. Ovine fetuses were exposed intra-amniotically (IA) to live *Ureaplasma parvum* 42 days (chronic stimulus) and/or to lipopolysaccharide (LPS) 2 or 7 days (acute stimulus) prior to preterm delivery at 124 days of gestational age (dGA). LPS exposure occurred at two different time points, since historical data report increased inflammatory and injurious pattern, upon treatment at respectively, 2 and 7 days before preterm delivery in the preterm ovine lungs (16, 17).

## Materials and Methods

### Study Approval

Animal experiments were approved by the animal ethics committee of the University of Western Australia (Perth, Australia).

### Animal Experiments and Tissue Sampling

**Figure 1:**
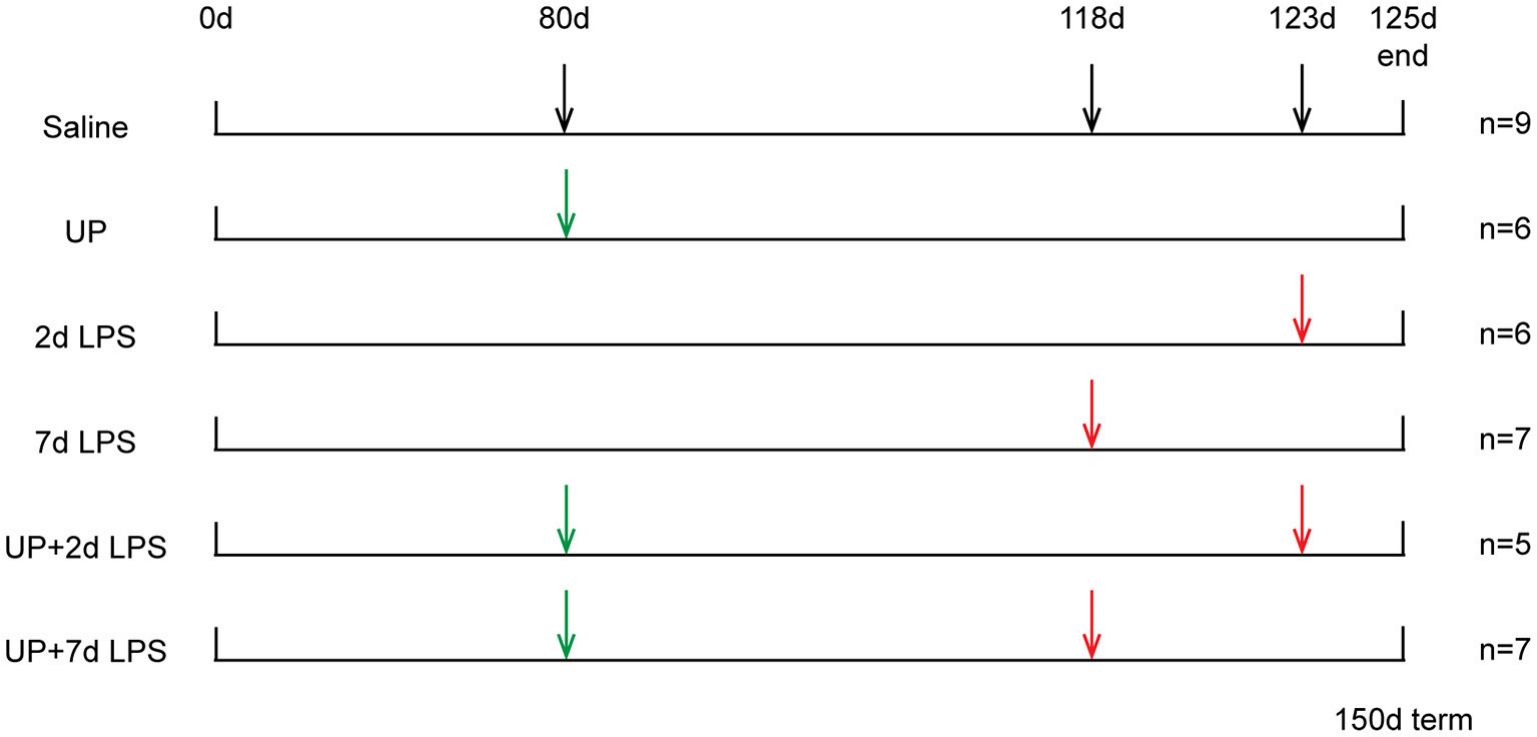
Study design of translational ovine model for antenatal stress. Ultrasound-guided intra-amniotic injections were used for the administration of saline, UP and LPS. Lambs were delivered preterm at 125d GA (150d term).

Procedures of the animal experiments and group allocations were published previously (10) and are presented in Figure 1. Study groups included 42 day exposure to UP (UP group), LPS exposure 2 or 7 days prior to preterm delivery (2d LPS, 7d LPS groups) and combined 42 days pre-exposure to UP and LPS exposure 2 or 7 days before preterm delivery at 125d GA (UP + 2d LPS, UP + 7d LPS groups). The 125d of GA in sheep correspond to the human gestation at ~31 weeks representing a moderate preterm neonate with a developing lung at the interface of the canalicular and saccular phase (18, 19).

Briefly, ultrasound-guided intra-amniotic injections were used to administer live UP serovar 3 strain HPA5 (Concentration: 2 × 10^5^ color-changing units CCU) and/or LPS (Concentration: 10 mg, *Escherichia coli* 055:B5; Sigma-Aldrich, St. Louis, MO) at pre-defined time points to 28 time-mated Merino ewes. Therefore, stock cultures of UP were diluted first in sterile culture medium and further in sterile saline (1:100). Sterile saline was also used for the dissolution of LPS and served as a comparable injection in control animals. After surgical delivery of the fetus, both, the ewe and the fetus were euthanized.

Pulmonary pressure volume assessment was conducted after euthanasia. Hereto, an endotracheal tube was introduced into the trachea and the thoracic cavity was opened to allow expansion of the lungs. The inflation of the lungs was achieved with air to a maximum pressure of 40 cm H_2_O. Lung deflation volumes were recorded at decreasing pressures starting at 40 cm H_2_O. Lung volumes were corrected for the body weight of the fetus (20).

Lung tissue sampling included inflation-fixation of the right upper lobe (RUL) for 24 h with 10% buffered formalin and snap freezing of the right lower lobe (RLL). The whole left lung was used to obtain bronchial lavage fluid.

### Histology and Immunohistochemistry

RUL paraffin-embedded lung sections of 4 μm thickness were used for (immuno)histochemical analysis. Tissue sections were stained with hematoxylin and eosin (H&E) for histological evaluation. In addition, the following cellular markers were visualized: CD45 for hematopoietic cells (1:500, MCA2220GA, Biorad, Hercules, CA), PU.1 for differentiating monocytes (1:400, Santa Cruz Biotechnology, H0503), myeloperoxidase for neutrophils (MPO, 1:500, A-0398, Dako, Santa Clara, CA), tumor protein 63 for basal cells (P63, 1:8000, ab124762, Abcam), keratin 14 for differentiating basal cells (KRT-14, 1:1000, 905301, Biolegend, San Diego, CA), thyroid transcription factor-1 for Club and alveolar epithelial type (AEC) 2 cells (TTF-1, 1:8000, WRAB-1231, Seven Hills Bioreagents, Cincinnati, OH) and Ki67 for proliferation (1:1000, 15580, Abcam, Cambridge, UK) (7, 12, 16, 21). Immunohistochemical protocols were performed as previously published, while the PU.1 protocol was modified for optimal signal emission. Briefly, lung sections were deparaffinized in xylol and decreasing ethanol series. Blocking of endogenous peroxidase activity was achieved by incubating in 0.3% H_2_O_2_ in 1xPBS for 20 min. For antigen retrieval, lung sections were boiled 5 min in citrate buffer (pH 6.0). To prevent aspecific binding of antibodies, sections were incubated with 5% bovine serum albumin in 1xPBS for 30 min. The primary antibody, PU.1 in 0.1% BSA/1xPBS, was added and incubated over night at 4°C. Next, sections were incubated for 1 h with biotin-labeled secondary Swine-anti-Rabbit antibody (1:200, E0353, Dako) in 0.1% BSA/1xPBS. Vectastain ABC Elite kit (PK-6100, Bio-connect) was used for the enhancement of the anti-body specific signal for 30 min. Tissue visualization was performed with diaminobenzidine staining for 90 s, followed by a counterstaining with hematoxylin for 20 s. Sections were dehydrated and coverslipped.

### Immunohistochemical Analyses

Methods for the analyses of immunohistochemical experiments have been published previously (7). Results for P63+, KRT-14+ and TTF-1+ cells were presented as cells per bronchus ring of proximal or distal airways, respectively. TTF-1+ and Ki67+ cells in alveoli were depicted as cells per high power field (HPF).

A magnification of 200x was used for PU.1 quantification and five randomly chosen pictures of alveoli (area of interest) were taken with a light microscope (Leica DM2000, Rijswijk, the Netherlands) and the Leica Application Suite 3.7.0 software (Leica Microsystem, Wetzlar, Germany). Alveolar region included the alveolar walls, alveolar airspaces and perivascular space. Analyses are presented as cells per HPF.

The wall-to-lumen ratio was determined on H&E sections, whereby vessels accompanying terminal bronchioles and an external diameter of <50 μm were investigated at a magnification of 400x. Five randomly chosen vessels were used and the wall-to-lumen ratio was calculated as media wall thickness divided by the radius of the vessel lumen (12).

Mean linear intercepts (MLI) and vessel density were also examined on H&E sections. Hereby, five and 10 images were taken randomly throughout the alveolar region, respectively for the MLI and the vessel quantification. In both cases bronchi and vessels (>50 μm external diameter) were excluded.

For the MLI assessment the ImageJ software (ImageJ 1.52i software, Bethesda, MD, USA) was used and images were superimposed with a 50 × 50 μm transparent grid. On five horizontal lines the intersections of the alveolar wall with the grid lines were counted. The MLI was determined according to the formula MLI = 2 × (L_tot_/L_x_), whereby, L_tot_ is the total length of all five lines and L_x_ is the total amount of intersections counted (22). Results are presented as micrometer of alveolar size.

With regard to the vessel quantification, all vessels, which were not accompanying a bronchus and had an external diameter <50 μm were counted (23). Surface area of alveolar tissue was determined with the Leica QWin Pro V3.5.1 software (Leica Microsystem) and results are displayed as vessels per square millimeter.

For all immunohistochemical stainings, as well as lung gas volumes, wall-to-lumen ratio, MLI and vessel quantification, the control values were presented as median and depicted as dotted line in all figures. Additionally, individual control values are provided in the Supplementary Table 1.

### RNA Extraction and Real-Time PCR

Snap frozen RLL tissue was used for RNA isolation, transcribed and amplified for the following genes (12, 21, 24): interleukin (IL)−6, IL-8, SRY-related HMG-box (SOX)−2, SOX-9, surfactant proteins (SP) -A, -B, -C, -D, aquaporin (Aqp) 5, vascular endothelial growth factor (VEGF) -a, VEGF receptor (VEGFR)−2, Angiopoeitin (Ang)−1, tyrosine-protein kinase receptor (Tie)-2, ribosomal protein S15 (RPS15), Glyceraldehyde 3-phosphate dehydrogenase (GAPDH) and Human 14-3-3 protein zeta/delta (YWHAZ). Due to limited availability of lung tissue, mRNA analysis could not be performed for all animals (some experimental groups miss 1-2 animals). RT-PCR data were converted with the LinReg software and normalized to the Geomean of the housekeeping genes RPS15, GAPDH and YWHAZ. Mean fold changes were calculated with the saline control values set at one. For all RT-PCR results the control values were presented as median and depicted as dotted line in all figures.

### Statistical Analysis

A non-parametric analysis of variance (ANOVA) followed by the *post-hoc* analysis Dunn's Multiple Comparison Test and a significance threshold of *p* < 0.05 were used to determine the statistical significance of the results (17). Results are displayed as median and interquartile range (IQR). *P*-values between 0.05 and 0.1 were interpreted as biologically relevant, as described previously (17). Significant changes toward the control groups were presented with asterisks, while differences between the experimental groups were shown with bars and asterisks.

## Results

### LPS Exposure Increases Pulmonary Inflammation, Which Is Not Further Modulated by Pre-exposure to UP

**Figure 2:**
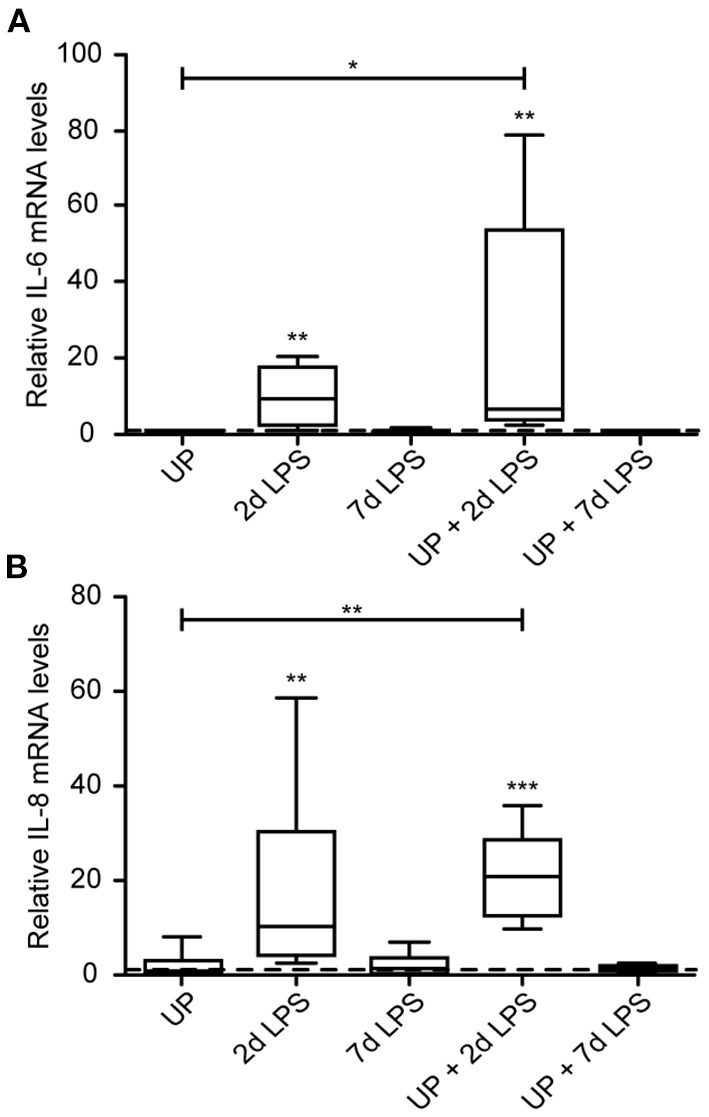
Cytokine and chemokine expression are increased after exposure to 2d LPS and UP + 2d LPS in preterm ovine lung tissue. Fold changes in mRNA levels for IL-6 **(A)** and IL-8 **(B)** are depicted against saline. The median saline value is represented as dotted line. *p < 0.05, **p < 0.01, ***p < 0.001 compared to saline and UP.

Single exposure to LPS resulted in increased immune activation when compared to control lambs, which was most pronounced in the 2d LPS-exposed lambs (Figure 2). In UP-exposed lambs, signs of increased inflammation were restricted to increased neutrophil infiltration. UP exposure prior to LPS exposure did not affect the observed increased expression of IL-6 and IL-8 after treatment with LPS alone.

**Figure 3:**
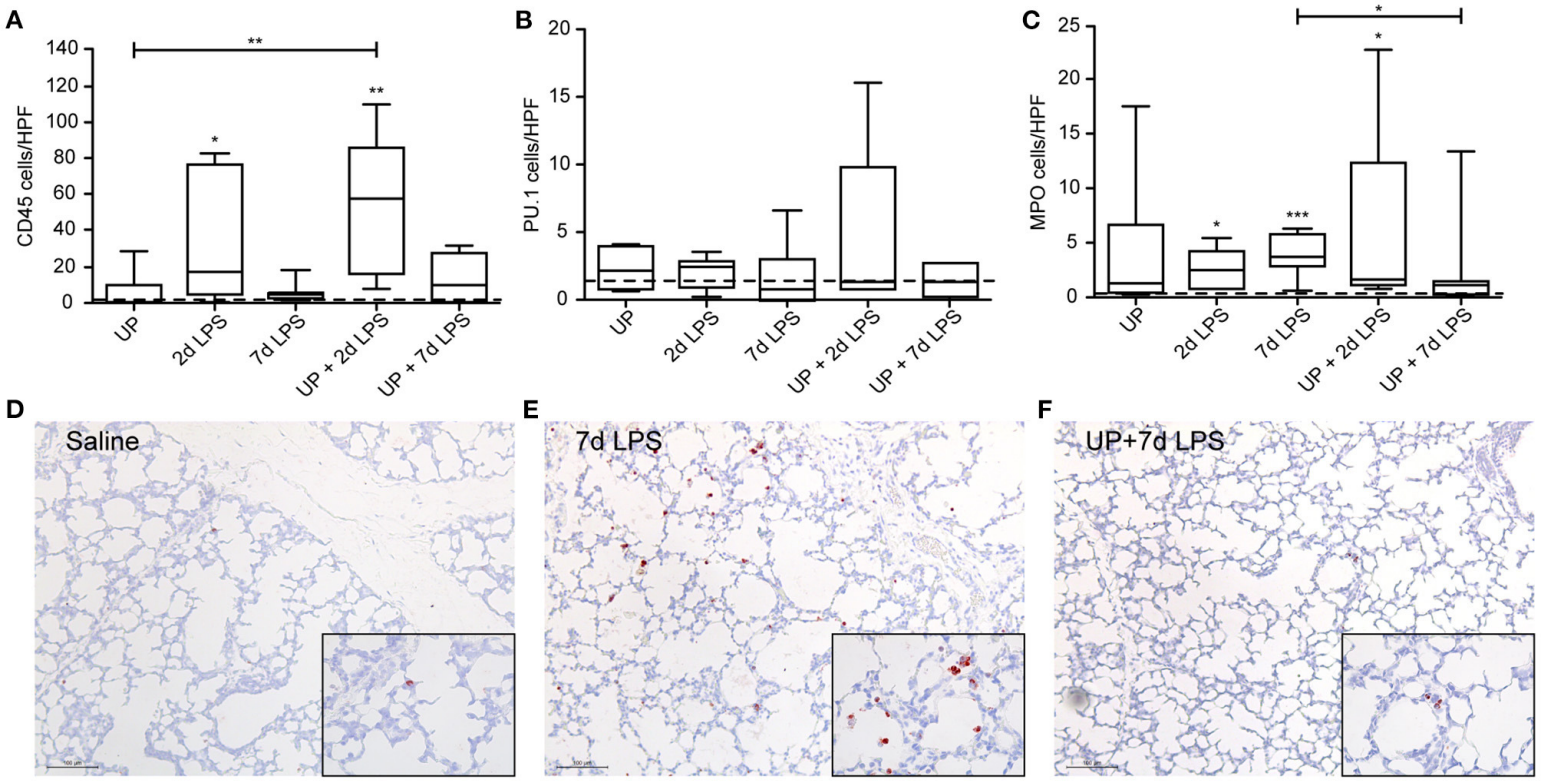
Single and sequential exposure induce immune cell infiltrations in the lung tissue, whereas neutrophil numbers are attenuated after UP + 7d LPS exposure. CD45+ immune cells **(A)**, PU.1+ macrophages **(B)** and MPO+ neutrophils **(C)** were quantified in alveoli and are presented as cells per HPF. The median saline value is represented as dotted line. Representative images are shown for MPO in saline **(D)**, 7d LPS **(E)** and UP + 7d LPS **(F)** groups. Image magnification is 200x, scale bar 100 μm. *p < 0.05, **p < 0.01, ***p < 0.001 compared to saline, UP and 7d LPS.

With regard to immune cell infiltration, the increased number of CD45+ immune cells reported in 2d LPS-exposed animals (Figure 3A), was also not affected by prior exposure to UP. Further, single and sequential inflammatory insults did not change the numbers of differentiating macrophages in the pulmonary tissue (Figure 3B). In addition, pre-exposure to UP did not significantly affect the increased number of neutrophils observed in the 2d LPS groups (Figure 3C). However, UP + 7d LPS showed that neutrophil numbers were attenuated to values similar to baseline levels (Figures 3D–F).

### Pre-exposure to UP Normalizes SOX-9 Expression, but Does Not Impact Stem/Progenitor Cell Responses, After an Initial Insult With LPS

**Figure 4:**
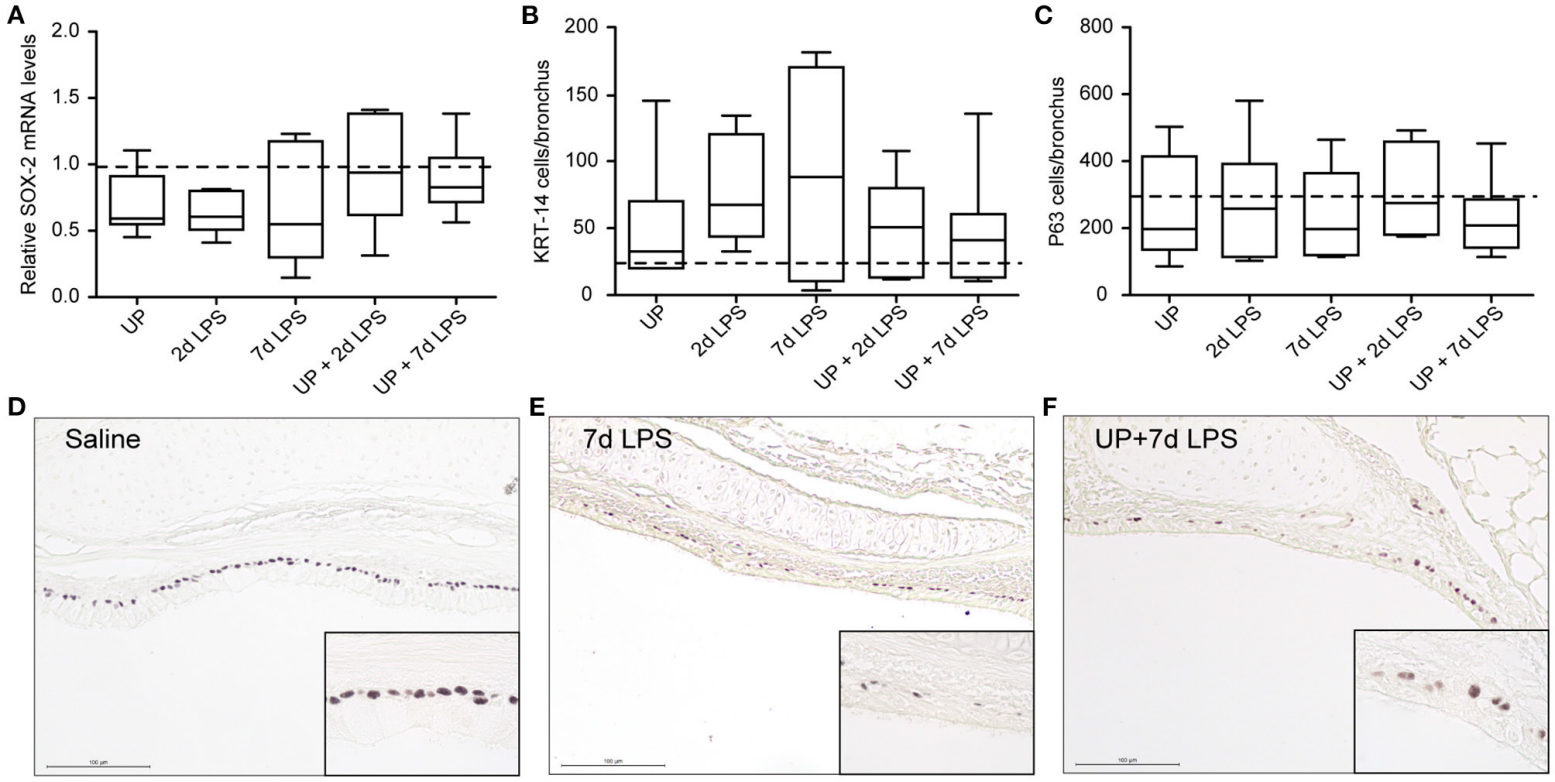
No further changes are observed in basal cells of the proximal airways after single and sequential antenatal inflammation. **(A)** SOX-2 fold changes in mRNA levels are depicted against saline. P63+ **(C)** and KRT-14+ **(B)** basal cells were quantified in proximal airways and are presented as cells per bronchus. The median saline value is represented as dotted line. Representative images are shown for P63 in saline **(D)**, 7d LPS **(E)** and UP + 7d LPS **(F)** groups. Image magnification is 200x, scale bar 100 μm.

Reduced numbers of endogenous stem/progenitor cell populations of the proximal airways have been reported after intra-amniotic exposure to LPS (7). Here, after sequential insults with UP and LPS no further reduction in P63+ and KRT-14+ cell numbers, as well as SOX-2 mRNA levels, were observed (Figure 4).

**Figure 5:**
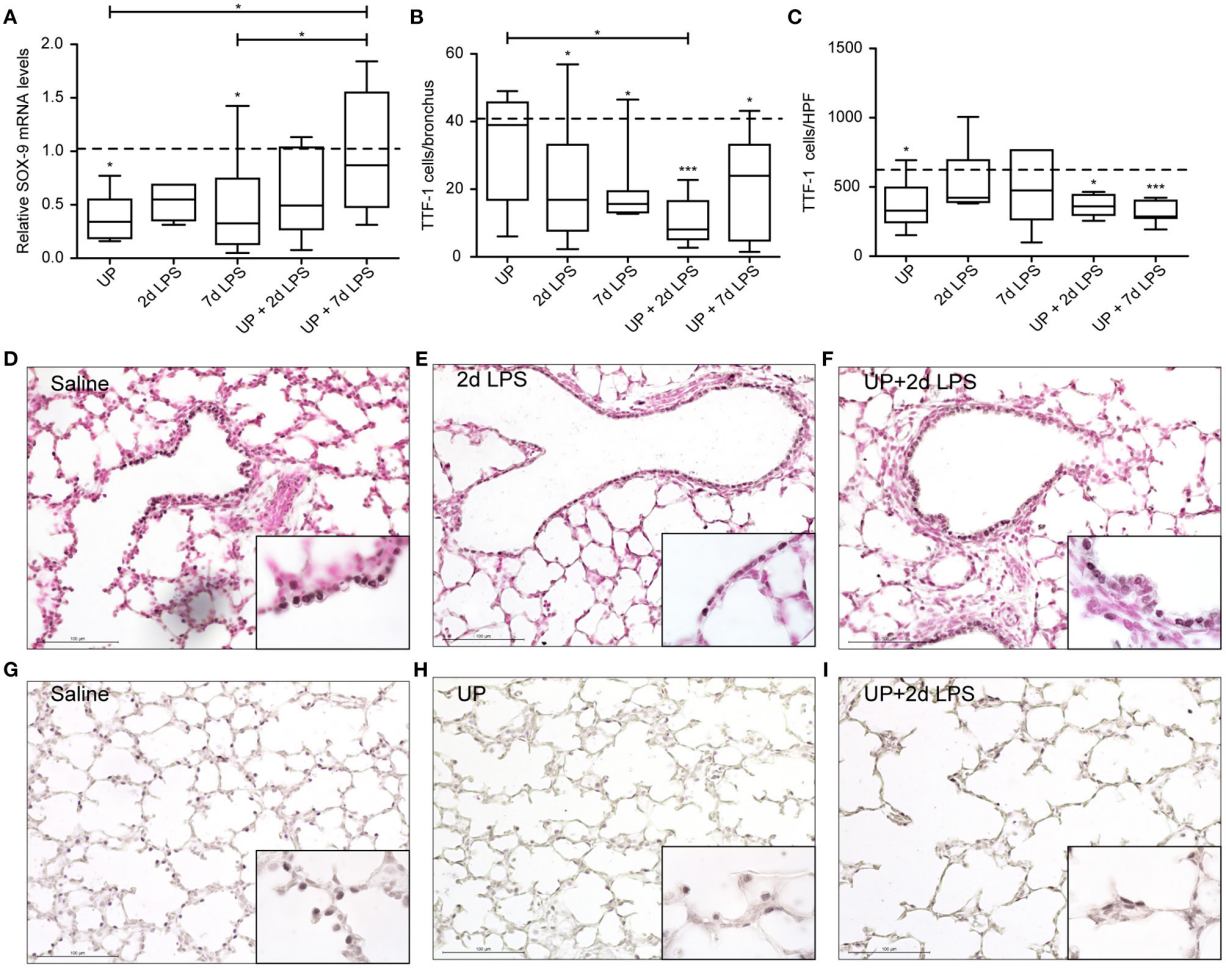
Pre-exposure to UP before the 7d LPS exposure normalizes SOX-9 expression but does not further impact stem/progenitor populations of the distal lung. **(A)** SOX-9 fold changes in mRNA levels are depicted against saline. TTF1+ **(B)** Club cells were quantified in distal airways and presented as cells per bronchus, while TTF-1+ **(C)** AEC2 were counted in alveoli and are presented as cells per HPF. The median saline value is represented as dotted line. Representative images are shown for Club cells in saline **(D)**, 2d LPS **(E)** and UP + 2d LPS **(F)** groups, and for AEC2 in saline **(G)**, UP **(H)** and UP+2d LPS **(I)** animals. Image magnification is 200x, scale bar 100 μm. *p < 0.05, ***p < 0.001 compared to saline, UP and 7d LPS.

In distal airways, UP and 7d LPS exposure alone decreased SOX-9 mRNA significantly (Figure 5A), while pre-exposure to UP in 7d LPS-exposed animals prevented a decrease in SOX-9 mRNA levels by a 3-fold increase in its expression. Club cell numbers were decreased in UP, 2d and 7d LPS groups, as well as in the UP + 2d LPS and UP + 7d LPS animals (Figures 5B,D–F). The number of AEC2 was significantly decreased in UP-infected animals, as well as in UP + 2d LPS and UP + 7d LPS animals compared to control (Figures 5C,G–I). Sequential exposure to UP and LPS did not further affect Club cells. In contrast, the most significant reduction in AEC2 numbers was observed in UP + 7d LPS-exposed animals.

### Prenatal Inflammation Affects Vascular Growth and Angiogenesis After Single Insults and UP Pre-exposure Sensitizes Vascular Disturbances to a Second Inflammatory Insult, Resulting in a Lower Vascular Density

Vascular remodeling is a common hallmark of BPD and has been found in models of pre- and postnatal inflammation. Here we assessed if single as well as sequential exposure induce vascular changes in preterm ovine lungs.

**Figure 6:**
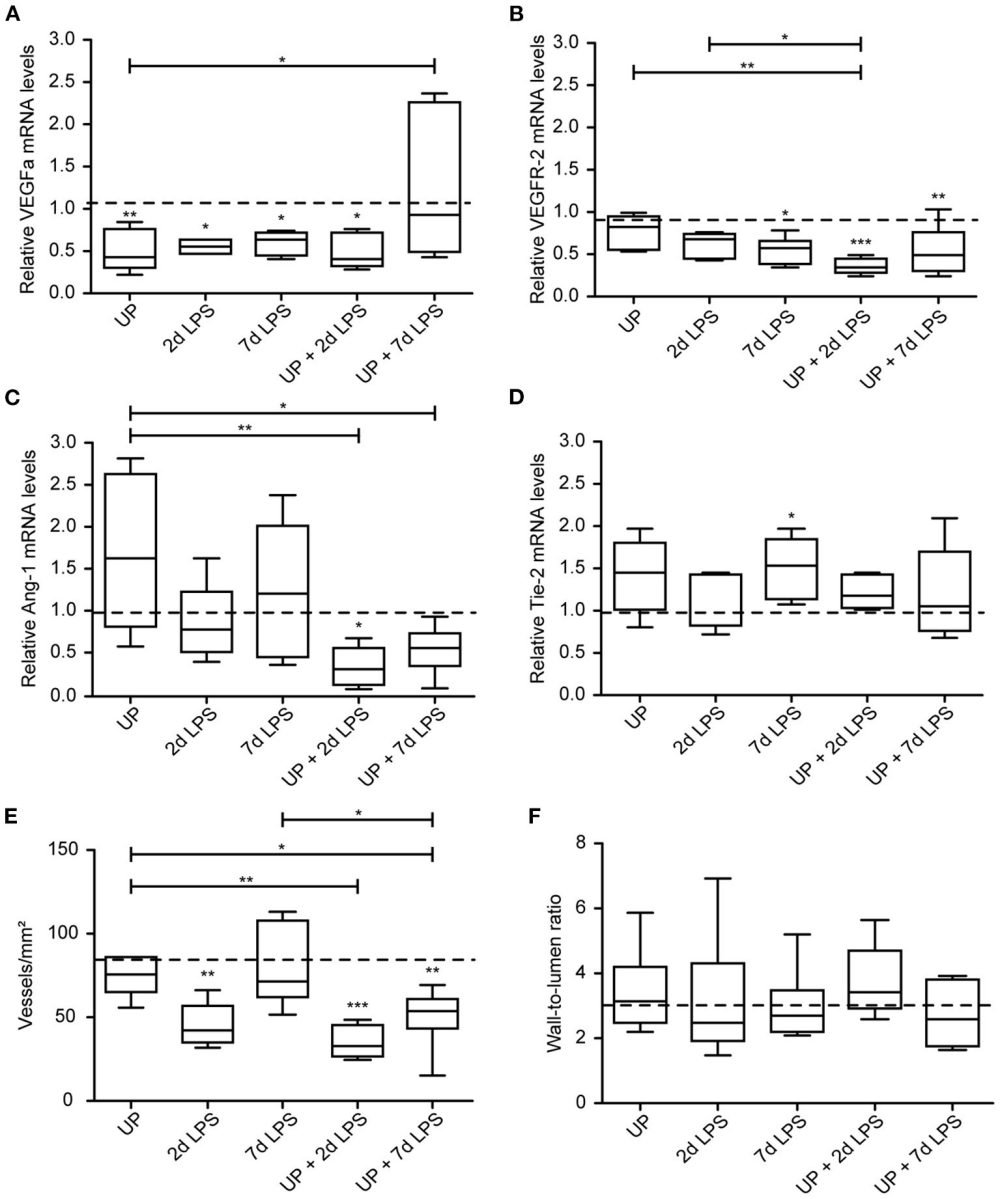
Sequential exposure to UP sensitizes vascular marker VEGFR-2 and Ang-1 to secondary LPS exposure and reduces the vascular density in alveolar tissue. VEGFa **(A)**, VEGFR-2 **(B)**, Ang-1 **(C)** and Tie-2 **(D)** fold changes in mRNA levels are depicted against saline. **(E)** Vascular quantification (for vessels <50 μm diameter) was performed and corrected for surface area of alveolar tissue. **(F)** Wall-to-lumen ratio was measured and calculated from small vessels (<50 μm diameter). The median saline value is represented as dotted line. *p < 0.05, **p < 0.01, ***p < 0.001 compared to saline, UP and 2d LPS.

Vascular development and angiogenesis were influenced by prenatal inflammation as evidenced by a significant drop in mRNA levels of VEGFa after single exposure to UP or LPS (Figure 6A). Pre-exposure to UP similarly decreased mRNA levels of VEGFa in the UP + 2d LPS group, whereas the mRNA levels were normalized to control in the combined UP + 7d LPS group. VEGFR-2 mRNA levels were significantly reduced in 7d LPS and UP + 7d LPS-exposed animals (Figure 6B). Although mRNA levels of VEGFR-2 were unaffected in the 2d LPS group, they were significantly lower in the UP + 2d LPS group compared to control, UP and 2d LPS-exposed animals. Ang-1 mRNA levels were not changed by single exposure to chronic or acute triggers, but showed a significant drop in both sequential exposure groups (Figure 6C). Tie-2 mRNA levels were increased in UP and 7d LPS groups and were unaffected after sequential exposure (Figure 6D).

These changes on mRNA level of the studied vascular and angiogenic markers prompted us to determine vessel density in the alveolar walls. While UP exposure alone did not alter the density of vessels, 2d LPS exposure decreased the number of vessels significantly by half compared to the control (Figure 6E). Pre-exposure to UP before (2 and 7d) LPS attenuated the vascular density even more prominently than LPS exposure alone.

Although changes in vascular and angiogenic markers were detected and the density of vessel reduced after prenatal inflammation, no alterations were measured in the wall-to-lumen ratio of the vessels by single or sequential inflammatory insults (Figure 6F).

### Developmental Alterations Found in Alveoli Are Most Prominent in UP + 7d LPS Exposed Lambs

**Figure 7:**
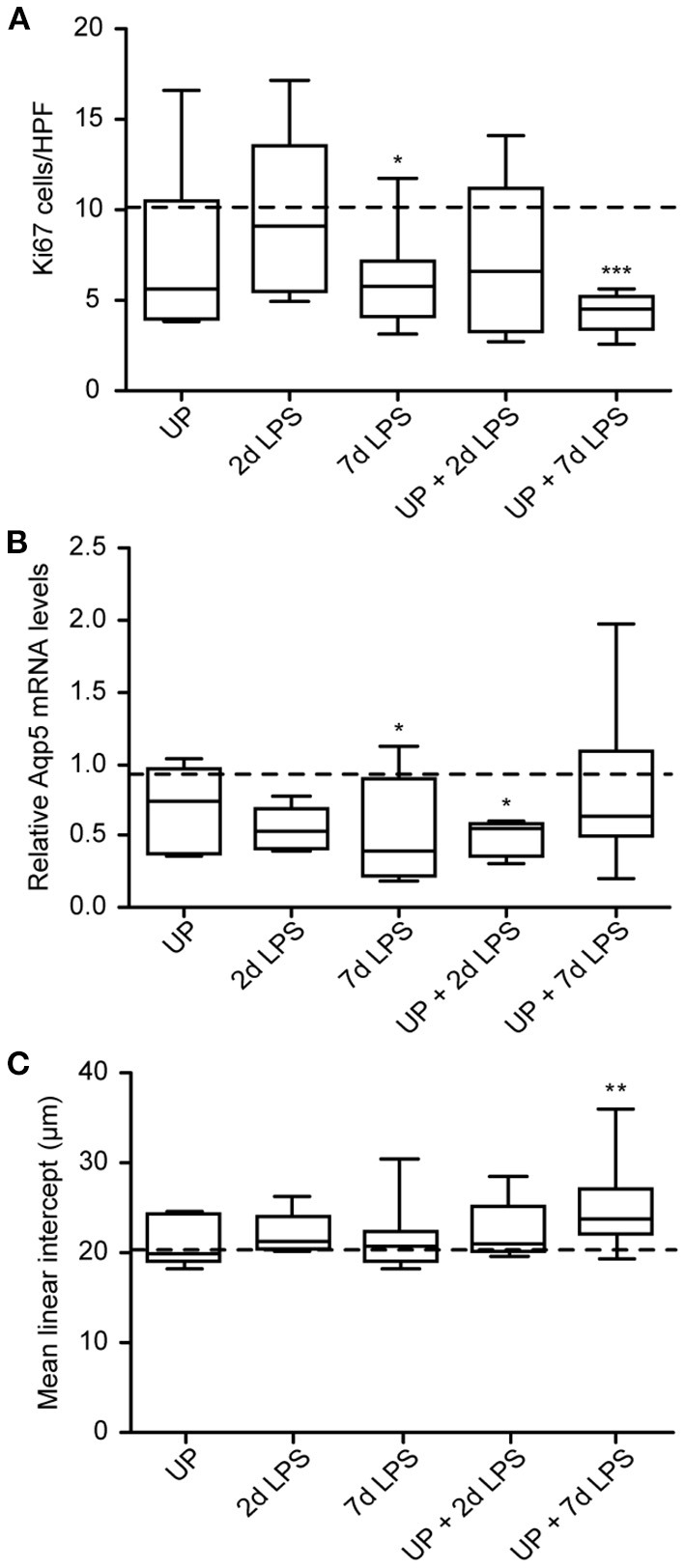
Developmental alveolar alterations are most prominent in UP + 7d LPS exposed lambs. **(A)** Ki67+ cells were quantified in alveoli and shown as cells per HPF. **(B)** Aqp5 fold changes in mRNA levels are depicted against saline. **(C)** The MLI was determined in alveolar tissue and represents the alveolar size in micrometer. The median saline value is represented as dotted line. *p < 0.05, **p < 0.01, ***p < 0.001 compared to saline.

Given the AEC2 alterations, we further assessed the alveolar development after sequential antenatal inflammation in terms of proliferation (Ki67) and differentiation (Aqp5) in the alveolar walls (Figure 7).

7d LPS and UP + 7d LPS exposure resulted in a significant drop to half of the amount of proliferating cells compared to the control group (Figure 7A). AEC1 were significantly decreased by half in the UP + 2d LPS group compared to control levels (Figure 7B). Additionally, while 7d LPS exposure decreased Aqp5 mRNA levels, sequential exposure did not result in a significant drop. With regard to the MLI, UP, and LPS exposure alone did not impact alveolar growth, whereas the exposure to UP followed by LPS 7 days before delivery increased the MLI. This result is consistent with the more significant decreased proliferation in the UP + 7d LPS group and the lower number of AEC2 in the same group when compared to single exposure with LPS (Figure 7C).

### Sequential Exposure to UP and LPS Has Additional Impact on mRNA Levels of Surfactant Proteins Compared to Single Inflammatory Insults, but Does Not Affect Lung Mechanics

As stem/progenitor cell numbers dropped in the distal lung compartments and sensitization of vascular signaling was observed, we further assessed the effects of sequential antenatal insults on functional parameters, including surfactant synthesis and lung mechanics (static lung compliance).

**Figure 8:**
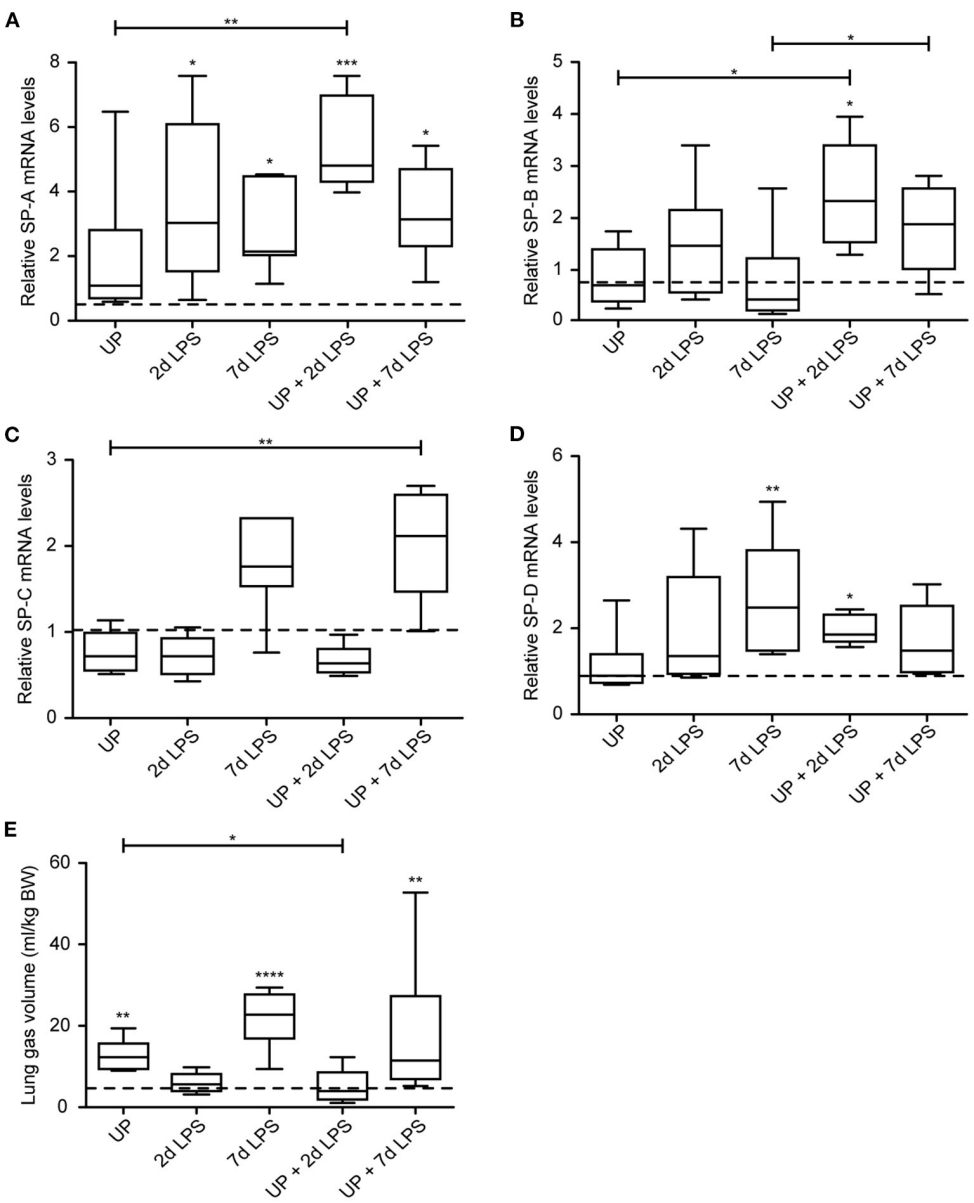
Sequential exposure to UP normalizes SP-B expression after 7d LPS. SP-A **(A)**, SP-B **(B)**, SP-C **(C)** and SP-D **(D)** fold changes in mRNA levels are depicted against saline. **(E)** Lung gas volumes of a pressure of 40 cm H_2_O are corrected for the bodyweight of the fetus and shown as ml/kg. The median saline value is represented as dotted line. *p < 0.05, **p < 0.01, ***p < 0.001, ****p < 0.0001 compared to saline, UP and 7d LPS.

While chronic UP exposure did not alter surfactant mRNA levels, we did see that 2d, 7d LPS groups, as well as pre-exposure with UP + 2d and +7d LPS caused an increase in mRNA levels of SP-A compared to controls and UP exposure (Figure 8A). UP pre-exposure pre-conditioned to 2d and 7d LPS exposure and thereby significantly increased mRNA levels of SP-B, whereas UP, 2d or 7d LPS groups did not result in mRNA changes (Figure 8B).

mRNA levels of SP-C were significantly increased in UP + 7d LPS groups, as were mRNA levels for single 7d LPS exposure (Figure 8C).

UP + 2d LPS exposure increased mRNA levels for SP-D compared to control and UP alone (Figure 8D). In contrast, UP + 7d LPS groups showed normalized SP-D mRNA levels compared to 7d LPS exposure alone.

Lung gas volumes were significantly increased at applied pressures of 0 cmH_2_O (data not shown) and 40 cmH_2_O in 7d LPS (10-fold) and UP + 7d LPS groups (4-fold) (Figure 8E). UP + 2d LPS exposure resulted in significantly lower lung gas volumes compared to UP alone.

## Discussion

There is increasing evidence that structural and functional abnormalities of the developing lungs that are provoked during pregnancy by inflammatory triggers, can contribute to postnatal lung pathology (25, 26). However, the mechanisms underlying these antenatal alterations remain largely unknown.

As an essential driver of lung development, endogenous epithelial stem/progenitor cells might play a role in prenatal maldevelopment of the lungs following inflammatory stressors (15, 27). Previously, we demonstrated fewer endogenous stem/progenitor populations as well as potential consequences thereof, including reduced alveolar differentiation, in the course of antenatal inflammation. Importantly, distinct stem cell changes in fetal ovine lungs were influenced by the timing and duration of a single chronic or acute inflammatory insult (7).

Clinically, perinatal organ development is frequently affected by multiple and repetitive inflammatory triggers, including infections, hypoxia, sepsis and mechanical ventilation, with serious consequences for the fetus/neonate. Clinical and pre-clinical studies have associated prenatal polymicrobial infections with a diversity of clinical outcomes (28, 29). This diversity in outcomes is difficult to estimate and therefore treatment of preterm infants might start too late to avoid serious postnatal problems. Apart from the microorganisms involved, investigating the effect of multiple inflammatory events during pregnancy is of great importance to understand their influences and impact on prenatal lung development. Prenatal infections, but also different maternal stressors and the event of birth are inevitable incidences that induce inflammation and that the fetus consequently has to cope with (30).

A prerequisite of studying multiple stressors is, to first determine the effects of the single components, which we have reported recently (7). In the current study, we extended our previous findings by investigating the effect of multiple sequential insults on important development processes in the preterm lungs and thereby increased the clinical relevance of this pre-clinical model. Moreover, we additionally examined consequences of single and multiple inflammatory events with respect to alveolar morphology and pulmonary vascular development in relation to altered stem/progenitor cell populations, mediators of vascular development and immunological changes.

Our study revealed that the strongest reduction of AEC2 and proliferating cells (Ki67+) was detected in lambs that were sequentially exposed to UP and 7d LPS. In line with this observation, decelerated alveolar growth was exclusively seen in this experimental group, indicated by increased MLI. Importantly, although single exposure to inflammatory stimuli did not result in significant morphological abnormalities, it negatively impacted epithelial stem/progenitor cell populations. These combined findings indicate that single inflammatory hits already negatively affect epithelial stem/progenitor cell populations including their function and numbers, a process that can be further aggravated when sequential inflammatory hits exert their negative effects synergistically. In this study, SOX-9 expression levels in the different experimental groups are of particular interest. SOX-9 expression is restricted to progenitor cells and disappears after proliferation and differentiation into different AEC2 subtypes (31, 32). The acquired single hit exposure data, which showed a reduction of SOX-9 expression in both the UP and LPS group, might potentially be responsible for reduced proliferation and reduced number of TTF-1+ AEC2 in developing alveoli. Of interest, consecutive hits with UP and (7d) LPS prevented a decrease in SOX-9 mRNA, while it caused the most pronounced reduction of TTF-1+ AEC2 and the number of proliferating cells, which was accompanied by an increased MLI. This finding potentially reflects a compensatory function for SOX-9 expression to counteract the reduced number of AEC2 with the pre-exposure to UP. It might be a timing effect that this compensatory function of SOX-9 did not initiate sufficient proliferation and differentiation yet to reverse the reduced number of AEC2. Such protective effects of SOX-9 have previously been observed in an acute lung injury (ALI) model, where SOX-9 was activated in the post-ALI phase and assumed to promote recovery of the damaged lungs (33). This scenario is currently investigated in ongoing postnatal studies. On the other hand, there are multiple transcription factors and developmental pathways involved in the complex process of distal lung development, which themselves potentially attenuated proliferation and growth of alveoli (34, 35). In this study, we also examined the pulmonary vasculature, due to its increasing importance in the development of BPD (36). Sequential inflammatory exposures negatively affected the growth and expansion of pulmonary vessels indicating that UP exposure primarily sensitizes animals that were subsequently exposed to 2d LPS. Consistent with this morphological observation, the pro-angiogenic and vascular factors, Ang-1 and VEGFR-2, were reduced by single inflammatory triggers and pre-exposure to UP sensitized these markers to a secondary insult with LPS. Moreover, VEGFa was decreased in all treatment groups, including this UP + 2d LPS group, which is further indicative for impaired vascularization. These current vascular changes after antenatal stress confirm and extend findings in a previous sequential hit study that was conducted at an earlier gestational age (94d GA in lambs, corresponding to extreme preterm infants in the canalicular stage of lung development) (12). These vascular disturbances, comprising decreased VEGFR-2 and Ang-1 mRNA levels after sequential exposure, seemed not affected by the GA of the fetuses, as comparable results were found in 94d and 125d GA fetuses. In addition, in both studies these disturbances were not associated with vascular remodeling in the preterm lungs (7, 12). Clinically, reduced and dysmorphic capillary networks have been reported in various BPD cohorts (37). Combined, the antenatal angiogenic data point toward a disturbed capillary network, which might be at the origin of postnatal adverse vascular development, an aspect that warrants further investigation (36).

In contrast to the attenuated alveolar growth and reduced vasculature, we observed a protective effect of sequential exposure with regard to surfactant synthesis, in particular SP-A and SP-B. Hereby increased SP-A and SP-B expression was found in UP + 2d LPS and UP + 7d LPS exposed animals, respectively, changes that were not as prominent in animals exposed to a single inflammatory trigger.

Our findings on SP-B expression are largely recapitulated by the observed inflammatory changes; Significant inflammatory changes were restricted to the number of neutrophils, which were attenuated in the 7d LPS group when they were pre-exposed to UP. It is tempting to speculate that this immune modulatory effect of UP, which has been described earlier by Kallapur et al. (13), might be involved in the protective effects on SP-B.

The essential role of SP-B in the survival of preterm infants at birth has been emphasized by various clinical studies (38). Chang et al. showed that a deficiency in SP-B through gene polymorphisms increased the risk to develop severe/lethal respiratory distress in preterm neonates (39, 40). Additionally, 75% of preterm infants with need for ventilation have been shown to have surfactant deficiencies in tracheal aspirates with 80% reduction in SP-B (41, 42). This key role of SP-B is attributed to its important function in the stabilization of the monolayer lipid films of surfactant, as well as in the absorption of lipids to the air/liquid interface (43, 44). Therefore, the increased SP-B mRNA levels, found after sequential insults with UP and LPS, might be an attempt to counteract the inflammation-driven changes, including decreased AEC2 numbers.

Besides this potentially protective effect of SP-B, also the increased expression of SP-A in UP pre-exposed animals (prior to 2d LPS) might indicate a beneficial effect. In the clinical situation and pre-clinical models, a deficiency in SP-A has been associated with an increased risk of BPD development in preterm neonates (24–29 week of GA) and immature baboons that received ventilation (45, 46). Additionally a reduced amount of SP-A mRNA has been reported in premature baboons with a BPD phenotype (47). These findings reveal a strong association between reduced SP-A proteins and BPD development. Through its important role in lung host defense, SP-A has been shown *in vitro* to promote increased ureaplasmacidal phagocytosis of UP isolates (from the BAL of premature infants with BPD) by murine macrophages (RAW 264.7) (48). Moreover, in *in vivo* studies, SP-A deficient mouse strains have been shown to display more excessive pulmonary inflammation after intra-tracheal UP administration compared to wild type controls. In these deficient SP-A mice also the clearance of UP occurred at a later time point (49). Taken together, the increased expression of SP-A after sequential exposure of UP and LPS could be interpreted as a means to eliminate UP from the preterm lungs.

Interestingly, these combined changes of increased SP-B and SP-A expression that suggest a protective effect by prior UP infection, did not overlap with improved lung function that was found to be significant in UP, 7d LPS, and UP + 7d LPS-exposed lambs. Moreover, this improved lung function was paralleled at the studied time points by detrimental alterations of stem/progenitor cells. This apparent lack of uniformity might be caused by a timing effect, but it most likely provides supporting evidence for the concept that perinatal inflammation improves lung function at the expense of inducing peripheral lung abnormalities, including decreased number of large and simplified alveoli, and abnormal pulmonary vascular development, predisposing to adverse postnatal pulmonary outcomes.

Besides the effects of consecutive inflammatory insults on lung development, also other immature organ systems are affected. The impact of multiple insults has been investigated in the preterm brain and gastrointestinal system. In particular, inflammation in the brain was less pronounced in LPS-exposed lambs when they were pre-exposed to UP. Additionally, the protective effect of UP was associated with reduced epigenetic changes (10). Sequential exposure of UP and LPS *in utero* did not amplify injury in the gastrointestinal and the enteric nervous system, that was caused by single exposure to UP or LPS (11). Taken together, these studies reflect the diversity in organ responses and outcomes after exposure to different infectious triggers (50). Additionally, also timing and duration of antenatal inflammatory triggers play a crucial role in the susceptibility of other organs and cells. Close monitoring of antenatal infection and inflammation is necessary for optimal risk classification of postnatal organ outcomes (10).

Regardless, these observed *in utero* alterations in essential cell populations, developmental factors and pulmonary morphology might render the preterm lungs more susceptible to sequential postnatal insults. Previously, it was shown that postnatal hits, including mechanical ventilation and oxygen supplementation, resulted in a decreased differentiation and proliferation potential in isolated lung endogenous stem cells (51). Postnatal cohort studies, using BPD and RDS samples, have also shown that preterm birth combined with common clinical practices, like oxygen supplementation and ventilation, resulted in decreased AEC2 and Club cell numbers, positive for TTF-1 (52, 53). Similarly, vascular abnormalities are a key hallmark of BPD and have been shown to be driven by perinatal insults (14, 36, 37). In a hyperoxia-induced BPD rat model, a lower capillary density was associated with reduced expression of VEGF and VEGFR-2 (54). Reduced and disorganized capillary development has further been reported in baboon models for BPD after interventional series of ventilation and supplemental oxygen (55).

The ovine pre-clinical model, which resembles the human *in utero* situation very closely, enables investigation of developmental disturbances in a prenatal inflammatory setting. The relatively long gestation of sheep, in which developmental stages occur similar as in humans, enables the precise interference in these stages (18, 19). In addition, microbial exposure can be exactly timed and thereby clinical inflammatory settings (chronic and acute) can be mimicked accurately. Another benefit of this study was the use of clinically relevant microorganisms, such as UP. Although LPS is not a living microorganism and specific microorganism-related responses might be missed, this *Escherichia coli*-derived endotoxin is a potent inducer of inflammation and therefore used to mimic clinical situations of acute inflammation. LPS responses are well-defined and accordingly less heterogenicity in responses is detected (56, 57).

Besides the advantages of the model and study, there are also some limitations. In the current study epithelial stem/progenitor populations have been investigated with the use of basic stem cells markers (P63, KRT-14, and TTF-1). However, the observed disturbances might be unique and restricted to a specific subpopulation. Additional examination with more extended techniques, such as cell sorting by FACS and single cell sequencing, would be informative to better define and understand the response of such subpopulations of stem/progenitor cells in the context of prenatal inflammation. Furthermore, considering the observed disturbances in vascular modulators, future investigations should include examination of endothelial stem/progenitor cell alterations. Moreover, fixed time points of intra-amniotic exposure to Ureaplasma or LPS were used, which did not enable us to dissect the effects of prenatal inflammation on extremely, moderate and late preterm organs. Importantly, the postnatal consequences of the observed *in utero* stem/progenitor cell changes are currently addressed in a postnatal follow up study.

Consistent with previous findings from our group and others, endogenous epithelial stem/progenitor cell populations are attenuated by perinatal inflammatory triggers (7, 58). Additionally, single inflammatory hits during pregnancy are also known to impair vascular growth (59). In the current study, we extended these findings by investigating the effects of sequential antenatal insults on alveolar growth and vascular maturation. We showed that exposure to a single inflammatory trigger already negatively impacts epithelial stem/progenitor cell populations including their function and numbers. This process was further aggravated by re-exposure to an inflammatory stimulus, resulting in disturbed alveolarization and abnormal pulmonary vascular development. The question whether these negative effects on lung development can be rescued by the potentially protective responses observed, will be addressed in an ongoing postnatal study.

Collectively, our data indicate that the type, timing and duration of antenatal stress determine the pulmonary outcome during pregnancy in the context of antenatal infections. Importantly, responses within the lungs can vary between lung compartment and cell types. Unraveling and linking the impact of antenatal and postnatal insults on the preterm lungs is of great importance to expand our understanding of the complex and multifactorial nature of BPD.

## Data Availability Statement

The original contributions generated for this study are included in the article/supplementary material, further inquiries can be directed to the corresponding author/s.

## Ethics Statement

The animal study was reviewed and approved by the animal ethics committee of the University of Western Australia.

## Author Contributions

HW, NR, and TW conceived and designed the research questions. MK, JN, MS, HU, MP, AJ, and BK designed and performed the *in vivo* study. HW conducted experiments, acquired and analyzed data. HW, NR, BK, TD, and TW contributed to the interpretation of results and drafted the manuscript. HW, NR, DO, MH, PN, CS-R, BK, TD, and TW edited and revised the manuscript. HW, NR, DO, MH, PN, CS-R, JC, MK, JN, MS, HU, MP, AJ, BK, TD, and TW read and approved final version of manuscript. All authors contributed to the article and approved the submitted version.

## Conflict of Interest

The authors declare that the research was conducted in the absence of any commercial or financial relationships that could be construed as a potential conflict of interest.

## References

[1] Malleske DT Chorna O Maitre NL. Pulmonary sequelae and functional limitations in children and adults with bronchopulmonary dysplasia. Paediatr Respir Rev. (2018) 26:55–9. 10.1016/j.prrv.2017.07.002 29031795

[2] Kallapur SG Jobe AH. Perinatal events and their influence on lung development and injury. In: The Newborn Lung. Elsevier (2019). p. 31–64. 10.1016/B978-0-323-54605-8.00002-7

[3] Jobe AH. The new bronchopulmonary dysplasia. Curr Opin Pediatr. (2011) 23:167–72. 10.1097/MOP.0b013e3283423e6b 21169836 PMC3265791

[4] Yee M Domm W Gelein R Bentley KL Kottmann RM Sime PJ. Alternative progenitor lineages regenerate the adult lung depleted of alveolar epithelial type 2 cells. Am J Respir Cell Mol Biol. (2017) 56:453–64. 10.1165/rcmb.2016-0150OC 27967234 PMC5449509

[5] Mandell EW Abman SH. Fetal vascular origins of bronchopulmonary dysplasia. J Pediatr. (2017) 185:7–10 e11. 10.1016/j.jpeds.2017.03.024 28359535

[6] Taglauer E Abman SH Keller RL. Recent advances in antenatal factors predisposing to bronchopulmonary dysplasia. Semin Perinatol. (2018) 42:413–24. 10.1053/j.semperi.2018.09.002 30389227 PMC6286866

[7] Widowski H Ophelders D van Leeuwen A Nikkels PGJ Severens-Rijvers CAH LaPointe VLS. Chorioamnionitis induces changes in ovine pulmonary endogenous epithelial stem/progenitor cells in utero. Pediatr Res. (2020) 1–11. 10.1038/s41390-020-01204-9 33070161

[8] Yoon BH Romero R Lim JH Shim SS Hong JS Shim JY. The clinical significance of detecting Ureaplasma urealyticum by the polymerase chain reaction in the amniotic fluid of patients with preterm labor. Am J Obstet Gynecol. (2003) 189:919–24. 10.1067/S0002-9378(03)00839-1 14586326

[9] Kallapur SG Presicce P Rueda CM Jobe AH Chougnet CA. Fetal immune response to chorioamnionitis. Semin Reprod Med. (2014) 32:56–67. 10.1055/s-0033-1361823 24390922 PMC4118297

[10] Gussenhoven R Ophelders D Kemp MW Payne MS Spiller OB Beeton ML. The paradoxical effects of chronic intra-amniotic *Ureaplasma parvum* exposure on ovine fetal brain development. Dev Neurosci. (2017) 39:472–86. 10.1159/000479021 28848098 PMC5828963

[11] Heymans C de Lange IH Hutten MC Lenaerts K de Ruijter NJE Kessels L. Chronic intra-uterine *Ureaplasma parvum* infection induces injury of the enteric nervous system in ovine fetuses. Front Immunol. (2020) 11:189. 10.3389/fimmu.2020.00189 32256485 PMC7089942

[12] Willems MG Kemp MW Fast LA Wagemaker NM Janssen LE Newnham JP. Pulmonary vascular changes in extremely preterm sheep after intra-amniotic exposure to *Ureaplasma parvum* and lipopolysaccharide. PLoS ONE. (2017) 12:e0180114. 10.1371/journal.pone.0180114 28666032 PMC5493356

[13] Kallapur SG Kramer BW Knox CL Berry CA Collins JJ Kemp MW. Chronic fetal exposure to *Ureaplasma parvum* suppresses innate immune responses in sheep. J Immunol. (2011) 187:2688–95. 10.4049/jimmunol.1100779 21784974 PMC3159703

[14] Alvira CM. Aberrant pulmonary vascular growth and remodeling in bronchopulmonary dysplasia. Front Med (Lausanne). (2016) 3:21. 10.3389/fmed.2016.00021 27243014 PMC4873491

[15] Collins JJ Thébaud BJBDRPAC Teratology M. Progenitor cells of the distal lung and their potential role in neonatal lung disease. Birth Defects Res A Clin Mol Teratol. (2014) 100:217–26. 10.1002/bdra.23227 24619857

[16] Collins JJ Kuypers E Nitsos I Jane Pillow J Polglase GR Kemp MW. LPS-induced chorioamnionitis and antenatal corticosteroids modulate Shh signaling in the ovine fetal lung. Am J Physiol Lung Cell Mol Physiol. (2012) 303:L778–87. 10.1152/ajplung.00280.2011 22962010 PMC3517680

[17] Willems MG Ophelders DR Nikiforou M Jellema RK Butz A Delhaas T. Systemic interleukin-2 administration improves lung function and modulates chorioamnionitis-induced pulmonary inflammation in the ovine fetus. Am J Physiol Lung Cell Mol Physiol. (2016) 310:L1–7. 10.1152/ajplung.00289.2015 26519206

[18] Pringle KC. Human fetal lung development and related animal models. Clin Obstet Gynecol. (1986) 29:502–13. 10.1097/00003081-198609000-00006 3757332

[19] Kramer BW. Chorioamnionitis - new ideas from experimental models. Neonatology. (2011) 99:320–5. 10.1159/000326620 21701204

[20] Jobe AH Newnham JP Willet KE Moss TJ Gore Ervin M Padbury JF. Endotoxin-induced lung maturation in preterm lambs is not mediated by cortisol. Am J Respir Crit Care Med. (2000) 162:1656–61. 10.1164/ajrccm.162.5.2003044 11069792

[21] Kuypers E Collins JJ Kramer BW Ofman G Nitsos I Pillow JJ. Intra-amniotic LPS and antenatal betamethasone: inflammation and maturation in preterm lamb lungs. Am J Physiol Lung Cell Mol Physiol. (2012) 302:L380–9. 10.1152/ajplung.00338.2011 22160306 PMC3289264

[22] Tschanz SA Makanya AN Haenni B Burri PH. Effects of neonatal high-dose short-term glucocorticoid treatment on the lung: a morphologic and morphometric study in the rat. Pediatr Res. (2003) 53:72–80. 10.1203/00006450-200301000-00014 12508084

[23] Moreira A Winter C Joy J Winter L Jones M Noronha M. Intranasal delivery of human umbilical cord Wharton's jelly mesenchymal stromal cells restores lung alveolarization and vascularization in experimental bronchopulmonary dysplasia. Stem Cells Transl Med. (2020) 9:221–34. 10.1002/sctm.18-0273 31774626 PMC6988765

[24] Atik A Sozo F Orgeig S Suri L Hanita T Harding R. Long-term pulmonary effects of intrauterine exposure to endotoxin following preterm birth in sheep. Reprod Sci. (2012) 19:1352–64. 10.1177/1933719112450327 22895023

[25] Gras-Le Guen C Denis C Franco-Montoya M-L Jarry A Delacourt C Potel G. Antenatal infection in the rabbit impairs post-natal growth and lung alveolarisation. Eur Respir J. (2008) 32:1520–8. 10.1183/09031936.00023708 18684851

[26] Kramer BW Kallapur S Newnham J Jobe AH. Prenatal inflammation and lung development. Semin Fetal Neonatal Med. (2009) 14:2–7. 10.1016/j.siny.2008.08.011 18845493 PMC2652840

[27] Leibel S Post M. Endogenous and exogenous stem/progenitor cells in the lung and their role in the pathogenesis and treatment of pediatric lung disease. Front Pediatr. (2016) 4:36. 10.3389/fped.2016.00036 27148506 PMC4830813

[28] Pammi M Zhong D Johnson Y Revell P Versalovic J. Polymicrobial bloodstream infections in the neonatal intensive care unit are associated with increased mortality: a case-control study. BMC Infect Dis. (2014) 14:390. 10.1186/1471-2334-14-390 25022748 PMC4226990

[29] Yoneda N Yoneda S Niimi H Ueno T Hayashi S Ito M. Polymicrobial amniotic fluid infection with mycoplasma/ureaplasma and other bacteria induces severe intra-amniotic inflammation associated with poor perinatal prognosis in preterm labor. Am J Reprod Immunol. (2016) 75:112–25. 10.1111/aji.12456 26668114

[30] Mulder EJ Robles de Medina PG Huizink AC Van den Bergh BR Buitelaar JK Visser GH. Prenatal maternal stress: effects on pregnancy and the (unborn) child. Early Hum Dev. (2002) 70:3–14. 10.1016/S0378-3782(02)00075-0 12441200

[31] Herriges M Morrisey EE. Lung development: orchestrating the generation and regeneration of a complex organ. Development. (2014) 141:502–13. 10.1242/dev.098186 24449833 PMC3899811

[32] Frank DB Penkala IJ Zepp JA Sivakumar A Linares-Saldana R Zacharias WJ. Early lineage specification defines alveolar epithelial ontogeny in the murine lung. Proc Natl Acad Sci U S A. (2019) 116:4362–71. 10.1073/pnas.1813952116 30782824 PMC6410851

[33] Li L Zhang H Min D Zhang R Wu J Qu H. Sox9 activation is essential for the recovery of lung function after acute lung injury. Cell Physiol Biochem. (2015) 37:1113–22. 10.1159/000430236 26402323

[34] Okubo T Knoepfler PS Eisenman RN Hogan BL. Nmyc plays an essential role during lung development as a dosage-sensitive regulator of progenitor cell proliferation and differentiation. Development. (2005) 132:1363–74. 10.1242/dev.01678 15716345

[35] Rawlins EL Clark CP Xue Y Hogan BL. The Id2+ distal tip lung epithelium contains individual multipotent embryonic progenitor cells. Development. (2009) 136:3741–5. 10.1242/dev.037317 19855016 PMC2766341

[36] Thebaud B Abman SH. Bronchopulmonary dysplasia: where have all the vessels gone? Roles of angiogenic growth factors in chronic lung disease. Am J Respir Crit Care Med. (2007) 175:978–85. 10.1164/rccm.200611-1660PP 17272782 PMC2176086

[37] Bhatt AJ Pryhuber GS Huyck H Watkins RH Metlay LA Maniscalco WM. Disrupted pulmonary vasculature and decreased vascular endothelial growth factor, Flt-1, and TIE-2 in human infants dying with bronchopulmonary dysplasia. Am J Respir Crit Care Med. (2001) 164:1971–80. 10.1164/ajrccm.164.10.2101140 11734454

[38] Fehrholz M Hutten M Kramer BW Speer CP Kunzmann S. Amplification of steroid-mediated SP-B expression by physiological levels of caffeine. Am J Physiol Lung Cell Mol Physiol. (2014) 306:L101–9. 10.1152/ajplung.00257.2013 24163141

[39] Nogee LM Garnier G Dietz HC Singer L Murphy AM deMello DE. A mutation in the surfactant protein B gene responsible for fatal neonatal respiratory disease in multiple kindreds. J Clin Invest. (1994) 93:1860–3. 10.1172/JCI117173 8163685 PMC294267

[40] Chang HY Li F Li FS Zheng CZ Lei YZ Wang J. Genetic polymorphisms of SP-A, SP-B, and SP-D and risk of respiratory distress syndrome in preterm neonates. Med Sci Monit. (2016) 22:5091–100. 10.12659/MSM.898553 28011976 PMC5207009

[41] Merrill JD Ballard RA Cnaan A Hibbs AM Godinez RI Godinez MH. Dysfunction of pulmonary surfactant in chronically ventilated premature infants. Pediatr Res. (2004) 56:918–26. 10.1203/01.PDR.0000145565.45490.D9 15496605

[42] Ballard PL Keller RL Truog WE Chapin C Horneman H Segal MR. Surfactant status and respiratory outcome in premature infants receiving late surfactant treatment. Pediatr Res. (2019) 85:305–11. 10.1038/s41390-018-0144-3 30140069 PMC6377352

[43] Veldhuizen EJ Haagsman HP. Role of pulmonary surfactant components in surface film formation and dynamics. Biochim Biophys Acta. (2000) 1467:255–70. 10.1016/S0005-2736(00)00256-X 11030586

[44] Nkadi PO Merritt TA Pillers DA. An overview of pulmonary surfactant in the neonate: genetics, metabolism, and the role of surfactant in health and disease. Mol Genet Metab. (2009) 97:95–101. 10.1016/j.ymgme.2009.01.015 19299177 PMC2880575

[45] Hallman M Merritt TA Akino T Bry K. Surfactant protein A, phosphatidylcholine, and surfactant inhibitors in epithelial lining fluid. Correlation with surface activity, severity of respiratory distress syndrome, and outcome in small premature infants. Am Rev Respir Dis. (1991) 144:1376–84. 10.1164/ajrccm/144.6.1376 1741552

[46] Awasthi S Coalson JJ Crouch E Yang F King RJ. Surfactant proteins A and D in premature baboons with chronic lung injury (Bronchopulmonary dysplasia). Evidence for an inhibition of secretion. Am J Respir Crit Care Med. (1999) 160:942–9. 10.1164/ajrccm.160.3.9806061 10471623

[47] Coalson JJ King RJ Yang F Winter V Whitsett JA Delemos RA. SP-A deficiency in primate model of bronchopulmonary dysplasia with infection. *In situ* mRNA and immunostains. Am J Respir Crit Care Med. (1995) 151:854–66. 10.1164/ajrccm/151.3_Pt_1.854 7881683

[48] Okogbule-Wonodi AC Chesko KL Famuyide ME Viscardi RM. Surfactant protein-A enhances ureaplasmacidal activity *in vitro*. Innate Immun. (2011) 17:145–51. 10.1177/1753425909360552 20197455

[49] Famuyide ME Hasday JD Carter HC Chesko KL He JR Viscardi RM. Surfactant protein-A limits Ureaplasma-mediated lung inflammation in a murine pneumonia model. Pediatr Res. (2009) 66:162–7. 10.1203/PDR.0b013e3181aabd66 19390477 PMC2758107

[50] Gantert M Been JV Gavilanes AW Garnier Y Zimmermann LJ Kramer BW. Chorioamnionitis: a multiorgan disease of the fetus? J Perinatol. (2010) 30:S21–30. 10.1038/jp.2010.96 20877404

[51] Moreira AG Siddiqui SK Macias R Johnson-Pais TL Wilson D Gelfond JAL. Oxygen and mechanical ventilation impede the functional properties of resident lung mesenchymal stromal cells. PLoS ONE. (2020) 15:e0229521. 10.1371/journal.pone.0229521 32142526 PMC7064315

[52] Stahlman MT Gray ME Whitsett JA. Expression of thyroid transcription factor-1 (TTF-1) in fetal and neonatal human lung. J Histochem Cytochem. (1996) 44:673–8. 10.1177/44.7.8675988 8675988

[53] Das I Das RN Paul B Mandal B Mukherjee S Chatterjee U. A study of spectrum of pulmonary pathology and expression of thyroid transcription factor-1 during neonatal period. Indian J Pathol Microbiol. (2018) 61:334. 10.4103/IJPM.IJPM_650_17 30004050

[54] Thebaud B Ladha F Michelakis ED Sawicka M Thurston G Eaton F. Vascular endothelial growth factor gene therapy increases survival, promotes lung angiogenesis, and prevents alveolar damage in hyperoxia-induced lung injury: evidence that angiogenesis participates in alveolarization. Circulation. (2005) 112:2477–86. 10.1161/CIRCULATIONAHA.105.541524 16230500

[55] Coalson JJ Winter VT Siler-Khodr T Yoder BA. Neonatal chronic lung disease in extremely immature baboons. Am J Respir Crit Care Med. (1999) 160:1333–46. 10.1164/ajrccm.160.4.9810071 10508826

[56] Raetz CR Whitfield C. Lipopolysaccharide endotoxins. Annu Rev Biochem. (2002) 71:635–700. 10.1146/annurev.biochem.71.110601.135414 12045108 PMC2569852

[57] Gilman-Sachs A Dambaeva S Salazar Garcia MD Hussein Y Kwak-Kim J Beaman K. Inflammation induced preterm labor and birth. J Reprod Immunol. (2018) 129:53–8. 10.1016/j.jri.2018.06.029 30025845

[58] Möbius MA Thébaud BJC. Bronchopulmonary dysplasia: where have all the stem cells gone?: origin and (potential) function of resident lung stem cells. Chest. (2017) 152:1043–52. 10.1016/j.chest.2017.04.173 28479114

[59] Kallapur SG Bachurski CJ Le Cras TD Joshi SN Ikegami M Jobe AH. Vascular changes after intra-amniotic endotoxin in preterm lamb lungs. Am J Physiol Lung Cell Mol Physiol. (2004) 287:L1178–85. 10.1152/ajplung.00049.2004 15321788

