# Supplementary material for: Sequential Exposure to Antenatal Microbial Triggers Attenuates Alveolar Growth and Pulmonary Vascular Development and Impacts Pulmonary Epithelial Stem/Progenitor Cells
Source: Front Med (Lausanne). 2021 Feb 22;8:614239. doi: 10.3389/fmed.2021.614239 (PMC7937719; doi:10.3389/fmed.2021.614239)
Supplement: Supplementary file 1 [file Table_1.DOCX]

| Group | Sample | CD45  cells/HPF | PU.1  cells/HPF | MPO  cells/HPF | P63  cells/bronchus | KRT14  cells/bronchus | TTF-1  cells/bronchus | TTF-1  cells/HPF | Ki67  cells/HPF | Vessel/mm2 | Wall-to-lumen ratio | MLI  µm | Lung gas  Volumes |
| --- | --- | --- | --- | --- | --- | --- | --- | --- | --- | --- | --- | --- | --- |
| SAL | 1 | 1 | 0,40 | 0 | 23 | 212 | 63 | 505 | 157 | 3,40 | 10 | 18,09 | 3,5 |
|  | 2 | 2,2 | 1,20 | 0 | 19 | 173 | 47 | 630 | 89 | 2,98 | 11 | 20,96 | 5,4 |
|  | 3 | 3,2 | 1,00 | 2 | 53 | 212 | 31 | 405 | 63 | 2,93 | 12 | 20,87 | 3,6 |
|  | 4 | 5,8 | 1,20 | 0 | 17 | 166 | 77 | 744 | 72 | 2,89 | 9 | 20,69 | 6,9 |
|  | 5 | 1,4 | 0,60 | 0 | 31 | 430 | 65 | 1072 | 83 | 2,49 | 7 | 21,06 | 4,90 |
|  | 6 | 1,4 | 3,60 | 0 | 115 | 293 | 33 | 669 | 138 | 2,99 | 12 | 18,36 | 0,00 |
|  | 7 | 1 | 0,40 | 0 | 16 | 383 | 35 | 416 | 92 | 3,38 | 6 | 20,69 | 5,80 |
|  | 8 | 0,6 | 13,00 | 0 | 26 | 430 | 42 | 915 | 51 | 2,66 | 10 | 20,11 | 3,90 |
|  | 9 | 0,6 | 1,60 | 2 | 85 | 454 | 36 | 591 | 77 | 3,49 | 13 | 15,89 | 4,50 |

Table 1. Individual results of the control groups for immunohistochemical stainings, vessel quantification, wall-to-lumen ratio, MLI and lung gas volumes.
